# A functional trait approach to identifying life history patterns in stochastic environments

**DOI:** 10.1002/ece3.5485

**Published:** 2019-07-31

**Authors:** Isabel M. Smallegange, Matty P. Berg

**Affiliations:** ^1^ Institute for Biodiversity and Ecosystem Dynamics (IBED) University of Amsterdam Amsterdam The Netherlands; ^2^ Department of Ecological Science, Section of Animal Ecology Vrije Universiteit Amsterdam The Netherlands; ^3^ Groningen Institute for Evolutionary Life Sciences, Community and Conservation Ecology Group Rijksuniversiteit Groningen Groningen The Netherlands

**Keywords:** body size, dynamic energy budget, ecosystem engineer, integral projection model, noise color, performance trait, salt marsh, stochastic demography, Talitridae

## Abstract

Temporal variation in demographic processes can greatly impact population dynamics. Perturbations of statistical coefficients that describe demographic rates within matrix models have, for example, revealed that stochastic population growth rates (log(*λ*_s_)) of fast life histories are more sensitive to temporal autocorrelation of environmental conditions than those of slow life histories. Yet, we know little about the mechanisms that drive such patterns. Here, we used a mechanistic, functional trait approach to examine the functional pathways by which a typical fast life history species, the macrodetrivore *Orchestia gammarellus*, and a typical slow life history species, the reef manta ray *Manta alfredi*, differ in their sensitivity to environmental autocorrelation if (a) growth and reproduction are described mechanistically by functional traits that adhere to the principle of energy conservation, and if (b) demographic variation is determined by temporal autocorrelation in food conditions. Opposite to previous findings, we found that *O. gammarellus* log(*λ*_s_) was most sensitive to the frequency of good food conditions, likely because reproduction traits, which directly impact population growth, were most influential to log(*λ*_s_). *Manta alfredi* log(*λ_s_*) was instead most sensitive to temporal autocorrelation, likely because growth parameters, which impact population growth indirectly, were most influential to log(*λ*_s_). This differential sensitivity to functional traits likely also explains why we found that *O. gammarellus* mean body size decreased (due to increased reproduction) but *M. alfredi* mean body size increased (due to increased individual growth) as food conditions became more favorable. Increasing demographic stochasticity under constant food conditions decreased *O. gammarellus* mean body size and increased log(*λ*_s_) due to increased reproduction, whereas *M. alfredi* mean body and log(*λ*_s_) decreased, likely due to decreased individual growth. Our findings signify the importance of integrating functional traits into demographic models as this provides mechanistic understanding of how environmental and demographic stochasticity affects population dynamics in stochastic environments.

## 1 INTRODUCTION

Unraveling the drivers of population growth in variable environments is critical for answering eco‐evolutionary questions (Ruokolainen, Linden, Kaitala, & Fowler, 2009; Smallegange, Deere, & Coulson, 2014; Tuljapurkar, 1982), developing conservation management strategies (Clark, 2010; Metcalf & Koons, 2007), predicting the time course of epidemics (Chaves, Scott, Morrison, & Takada, 2014; Keeling & Gilligan, 2000) and in influencing the yield of harvested populations (Cameron et al., 2016; Higgins, Hastings, Sarvela, & Botsford, 1997; Smallegange & Ens, 2018). Both demographic processes and temporal variation in environmental conditions are important drivers of population growth (Higgins et al., 1997; Lande, Engen, & Sæther, 2003; Turchin & Taylor, 1992). Typically, neither demography nor environmental conditions are solely responsible for driving population‐level responses (Lande et al., 2003; Shelton & Mangel, 2011), and research efforts, therefore, concentrate on identifying and understanding the population consequences of their interaction (Lande et al., 2003; Munch, Snover, Watters, & Mangel, 2005; Paniw, Ozgul, & Salguero‐Gómez, 2018).

Interactions between environmental conditions and demography arise because variation in environmental conditions translates into variation in the demographic rates of survival, growth and reproduction, thereby affecting population sizes (Boyce, Haridas, & Lee, 2006; Coulson et al., 2011). The dynamics of such change have been studied extensively in stochastic population analyses where temporal variation in environmental conditions lacks temporal autocorrelation (Getz & Haight, 1989; Lande, Sæther, & Engen, 1997). Results from these analyses reveal that long‐lived species with slow life histories, characterized by high survival and low fecundity (Gaillard et al., 1989), are generally buffered from increased environmental variation (Morris et al., 2008, Morris et al., 2011; Dalgleish, Koons, & Adler, 2010; Sæther et al., 2013; but see Jongejans, Kroon, Tuljapurkar, & Shea, 2010; McDonald et al., 2017). Short‐lived species with fast life histories characterized by high fecundity but low survival (Gaillard et al., 1989), on the other hand, are more likely to show increasing fluctuations in population sizes with increasing environmental variation (McDonald et al., 2017; Morris et al., 2008).

In nature, environmental fluctuations usually show positive autocorrelation over time (Ariño & Pimm, 1995; Halley, 1996; Inchausti & Halley, 2002). Yet, we are only beginning to unravel whether links between life history characteristics and population responses to environmental fluctuations identified for uncorrelated environments (Dalgleish et al., 2010; Morris et al., 2008) hold in biologically more realistic, autocorrelated environments. For example, in mite microcosm experiments, life stage responses to environmental perturbations (harvesting) differ between autocorrelated and uncorrelated environments (Cameron et al., 2016; Smallegange & Ens, 2018). Theoretical studies have shown that a change in the serial correlation of demographic rates may increase or decrease population growth rate depending on the structure of the life history (Tuljapurkar, Gaillard, & Coulson, 2009). Using a cross‐taxonomical approach, Paniw et al. (2018) most recently illustrated that species characterized by fast life histories exhibit the highest sensitivities to simulated autocorrelation in demographic rates, whereas slow life history species were less sensitive to temporal autocorrelation. With predicted global changes in environmental patterning (García‐Carreras & Reuman, 2011), it is urgent to gain a mechanistic understanding of the life history processes that result in these distinct demographic responses between slow and fast life histories to temporal autocorrelation in environmental conditions. The demographic approaches used to date to map out life history patterns across stochastic environments, however, lack a mechanistic representation of the biological processes that give rise to observed demographic variation (Salguero‐Gómez, Violle, Gimenez, & Childs, 2018), as the state variables used in the models (e.g., body size, developmental stage, age) are too phenomenological to explore underlying mechanisms of variation (Salguero‐Gómez, 2017).

Mechanistic trait‐based models can provide functional insights into the links between individual life history, the environment and population dynamics. Recently, demographic functions describing growth and reproduction have been derived from a simple dynamic energy budget (DEB) growth model, and incorporated into an integral projection model (IPM) (Easterling, Ellner, & Dixon, 2000) to describe survival, growth and reproduction in relation to the feeding environment (Smallegange, Caswell, Toorians, & Roos, 2017). Here we used such DEB‐IPMs to examine the functional pathways by which a typical fast and a typical slow life history species differ in their response to shifts in temporal autocorrelation when their individual growth and reproduction are described by functional, life history traits. We conducted a field study on a barrier island salt marsh to parameterize a DEB‐IPM for the fast‐reproducing and short‐lived macrodetritivore talitrid beach hopper (*Orchestia gammarellus*) and used a published DEB‐IPM of the slow‐reproducing, long‐lived reef manta ray (*Manta alfredi*) (Smallegange et al., 2017). This study is also a demonstration of how a unified method like the DEB‐IPM can be applied to species with extremely different life cycles. For each DEB‐IPM, we carried out stochastic simulations to assess if the fast (or slow) life history species showed high (or low) sensitivity of the stochastic population growth rate (log(*λ*_s_)) to shifts in temporal autocorrelation in food availability (Morris et al., 2008; Paniw et al., 2018). Because environmental effects can affect individual growth and thereby the size of individuals (DeLong et al., 2015; Woodward et al., 2005), we also explored patterns in mean body size across the variable environments. In the stochastic demographic model, the temporal sequence of good and bad food environments was driven by a Markovian process that governs the serial correlation of environment states. In order to cover a wide range of stochastic environments, we varied this serial correlation from blue to white to red noise color (corresponding to a negative first‐order autocorrelation, no autocorrelation, and a positive first‐order autocorrelation of the temporal sequence, respectively), but also varied the frequency at which the good environment occurred from zero to one. We also conducted a perturbation analysis for each species to assess which life history trait had the strongest effect on the stochastic population growth rate, as this mediates population‐level responses to temporal autocorrelation (Franco & Silvertown, 1996, 2004; Tuljapurkar & Haridas, 2006). Finally, we assessed to what extent patterns in log(*λ*_s_), mean body size and elasticities depended on demographic stochasticity (inherent randomness in demographic processes (Lande et al., 2003), for example, when individuals of the same size and in the same environment, independently of each other, have good or bad luck in their feeding experience), as demographic stochasticity, population structure and temporal autocorrelation can interact to shape population dynamics (Vindenes & Engen, 2017).

## 2 MATERIAL AND METHODS

### 2.1 Brief model description

If a female survives from time *t* to time *t* + 1, she grows from length *L* to length *L*′ following a von Bertalanffy growth curve. If a surviving female is an adult, she also produces eggs. These events are captured by four fundamental functions: the survival function, *S*(*L*(*t*)), the growth function, G(*L*′,*L*(*t*)), the reproduction function, *R*(*L*(*t*)), and the parent‐offspring function, *D*(*L*′,*L*(*t*)), which describes the association between the body length of the parent *L* and offspring length *L*′. Denoting the number of females at time *t* by *N*(*L*,*t*) means that the dynamics of this body length number distribution from time *t* to *t* + 1 can be written as:

(1) $$N\left(L^{\prime },t+1\right)=\int_{\Omega }\left[D\left(L^{\prime },L\left(t\right)\right)R\left(L\left(t\right)\right)+G\left(L^{\prime },L\left(t\right)\right)S\left(L\left(t\right)\right)\right]N\left(L,t\right)dL,$$

where the closed interval Ω denotes the length domain. The survival function *S*(*L*(*t*)) in Equation 1 is the probability that an individual of length *L* survives from time *t* to *t* + 1:

(2) $$S\left(L\left(t\right)\right)=\left\{\begin{array}{c} e^{-\mu }\;\mathrm{for}\;L\leq L_{m}E\left(Y\right)/\kappa \\ 0\;\text{otherwise} \end{array}\right.,$$

where *E*(*Y*) is the expected feeding level, which ranges from zero (empty gut) to one (full gut). Here, individuals that experience *E*(*Y*) = 1 can be assumed to always have a full gut. The parameter *L*_m_ is the maximum body length under conditions of unlimited resource, *E*(*Y*) = 1, and *κ* is the fraction of ingested energy allocated to respiration. Individuals die from starvation at a body length at which maintenance requirements exceed the total amount of assimilated energy, which occurs when $L>L_{m}\cdot E\left(Y\right)/\kappa$ and hence, then, *S*(*L*(*t*)) = 0 (e.g., an individual of size *L_m_* will die of starvation if *E*(*Y*) <* κ*).

The function G(*L*′,*L*(*t*)) is the probability that an individual of body length *L* at time *t* grows to length *L'* at *t* + 1, conditional on survival, and, following common practice (Easterling et al., 2000), follows a Gaussian distribution:

(3) $$G\left(L^{\prime },L\left(t\right)\right)=\frac{1}{\sqrt{2\pi \sigma_{L}\left(L\left(t+1\right)\right)}}e^{\frac{-(L^{\prime }-E{\left(L\left(t+1\right)\right)}^{2}}{2\sigma_{L}^{2}\left(L\left(t+1\right)\right)}}$$

with

(4) $$E\left(L\left(t+1\right)\right)=L\left(t\right)e^{-\overset{\cdot }{r_{B}}}+\left(1-e^{-\overset{\cdot }{r_{B}}}\right)L_{m}\cdot E\left(Y\right),$$

and

(5) $$\sigma^{2}\left(L\left(t+1\right)\right)={\left(1-e^{-\overset{\cdot }{r_{B}}}\right)}^{2}L_{m}^{2}\sigma^{2}\left(Y\right),$$

where $\overset{\cdot }{\underset{B}{r}}$ is known as the von Bertalanffy growth rate and *σ*(*Y*) is the standard deviation of the expected feeding level. Note that we assumed that individuals can shrink under starvation conditions.

The reproduction function *R*(*L*(*t*)) gives the number of offspring produced between time *t* and *t* + 1 by an individual of length *L* at time *t*:

(6) $$\begin{array}{c} R\left(L\left(t\right)\right)=\left\{\begin{array}{cc} 0 & \mathrm{for}\;L_{B}\leq L<L_{p}\\ E\left(Y\right)R_{m}L{\left(t\right)}^{2}/L_{m}^{2} & \mathrm{for}\;L_{p}\leq L\leq L_{m}E\left(Y\right)/\kappa \end{array}\right., \end{array}$$

where *R*_m_ is the maximum reproduction rate of an individual of maximum body length *L*_m_. Individuals are mature when they reach puberty at body length *L*_p_ and only surviving adults reproduce (Equation 1); thus, only individuals within a cohort of length *L*_p_ ≤ *L* ≤ *L*_m_ *Y*/*κ* reproduce.

The probability density function *D*(*L*′,*L*(*t*)) gives the probability that the offspring of an individual of body length *L* are of length *L'* at time *t* + 1, and hence describes the association between parent and offspring character values:

(7) $$\begin{array}{c} D\left(L^{\prime },L\left(t\right)\right)=\left\{\begin{array}{cc} 0 & \text{for}\;L<L_{p}\\ \frac{1}{\sqrt{2\pi \sigma_{L_{b}}\left(L\left(t\right)\right)}}e^{\frac{-\left(\right.L^{\prime }-E_{L_{b}}{\left(L\left(t\right)\right)}^{2}}{2\sigma_{L_{b}}^{2}\left(L\left(t\right)\right)}} & \text{otherwise} \end{array}\right. \end{array}$$

where $E_{L_{B}}\left(L\left(t\right)\right)$ is the expected size of offspring produced by a cohort of individuals with length *L*(*t*), and $\sigma_{L_{B}}^{2}\left(L\left(t\right)\right)$ the associated variance. For simplicity, we set $E_{L_{B}}\left(L\left(t\right)\right)$ constant and assumed its associated variance, $\sigma_{L_{B}}^{2}\left(L\left(t\right)\right)$, to be very small.

### 2.2 Field study to collect *O. gammarellus* life history data

**Figure 1:**
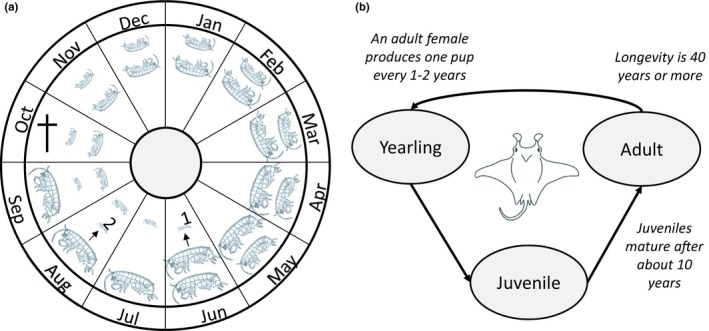
(a) Schematic representation of the life cycle of *Orchestia gammarellus*. The first cohort of juveniles is born between mid‐June and early July (arrow 1), depending on the temperature conditions in late winter and spring. Probably, the same females produce a second cohort of juveniles about 6 weeks later in early August (arrow 2). Then, the females that have reproduced die in October (cross), after having lived approximately for 1 year. (b) *Manta alfredi* life cycle. Adult females produce on pup per 1–2 years, which take on average 10 years to mature, after which individuals live for another 30 years or more

The litter feeder *O. gammarellus* is a terrestrial amphipod belonging to the family Talitridae (Figure 1a). It is a bioturbating soil animal that typically lives in the litter and soil surface layer of tall, salt marsh intermediate‐ and late‐successional‐stage vegetation (Schrama, Berg, & Olff, 2012). At our field site, the salt marsh of the barrier Island of Schiermonnikoog, the Netherlands (53°28′N and 6°12′E), the animals were found between 1.30 and 1.54 m above mean sea level. They tolerate regular submersion in saltwater due to their osmoregulatory capacity (Morrit, 1989). As is typical of most soil fauna, they are sensitive to high temperatures and drought (Moore & Francis, 1986a). In temperate regions, this species has a yearly life cycle with two reproductive periods, the first at mid‐June/early July and the second in early–mid‐August (Figure 1a). Both cohorts overwinter in the subadult or adult stage and reproduce in the summer the year after they were born. Mortality mainly occurs after the second reproductive peak. Talitridae do not lay eggs in the soil but carry eggs and embryos in a brood pouch between their legs, in which an average female carries about 17 eggs. Populations are typically female biased (Moore & Francis, 1986b), with on average four times as many females as males (M. P. Berg, unpublished data).

Measurements of O. gammarellus's life history and population size have been taken in the salt marsh of Schiermonnikoog since 2014. From 2015 to 2017, we have data on body size distributions (in terms of juvenile and adult mean body length), but only for 2017 we also have data on population structure (in terms of proportion of eggs, juveniles, and adults). The average annual air temperature on the island is 10.2°C (±0.72), and the average yearly rainfall is 824 mm (±149.1) (data obtained from the Royal Netherlands Meteorological Institute at www.knmi.nl). The study site was on a cattle‐ and geese‐grazed part of the salt marsh, at the late‐successional stage of 120 years of vegetation succession (Olff, Leeuw, Bakker, Platerink, & van Wijnen, 1997). The vegetation consisted of a mosaic of tall vegetation dominated by sea couch (*Elytrigia atherica*) and sea rush (*Juncus maritimus*) intermingled with short, grazed vegetation dominated by common saltmarsh grass (*Puccinellia maritima*), sea plantain (*Plantago maritima*), and sea milkwort (*Glaux maritima*). The sample site was selected at an intermediate salt marsh height (53°28′55N, 6°12′56E) along a low (close to the sea) to high (2 km away from the sea) elevational gradient, which had an average inundation frequency of 60 times per year. The soil consisted of a 12‐cm‐thick clay layer on bare sand with a base elevation of 1.16 m (±*SE* 2.2 cm above the Dutch Ordinance Level).

Every year, six to seven population samples were taken: once a month in summer, every 2 months in winter, and every 6 weeks in spring and autumn. Samples were taken from under old, partly dried‐out cow dung, which is a preferred microhabitat of *O. gammarellus*, using a plastic bucket (27 cm Ø) without a bottom. The bucket was placed around the dung and pressed 2 cm into the ground to ensure that the whole population was sampled. Typically, 100–750 specimens were collected per sample by hand and stored in 96% ethanol. For each sample, we measured the length (mm) of the first pereon segment and the number of podomeres in the antennal flagellum of all individuals. As the body of *O. gammarellus* is curved, it is difficult to measure total body length accurately. The first pereon overlaps the head and second pereon and can be measured reliably, and using the allometric relationship Body length=0.3797+11.38056·pereonlength (M. P. Berg, unpublished data), we estimated total body length *L*. The number of podomeres was used to estimate the developmental stage of the individuals. On average, one podomere is added to the flagellum with each moult (Wildish, 1969). Juveniles are born with four podomeres and females carrying eggs typically have 12 or more podomeres, while the oldest individuals have 21 podomeres as a maximum.

### 2.3 DEB‐IPM parameterization for *O. gammarellus*

We used *O. gammarellus* life history data collected from our field site (see above) to calculate input values for our model analyses. Mean body length at birth was measured at 3.80 ± 0.37 *SD* (standard deviation) mm. Females matured at a mean body length of 7.28 mm ± 0.50 *SD* mm and can reach a maximum length of 15.61 mm. Based on these observations, we set *L*_b_ = 3.80 mm, *L*_p_ = 7.28 mm and *L*_m_ = 15.61 mm. There are two reproduction peaks, one in mid‐June/early July and the second one in early–mid‐August; the highest number of offspring produced by a female in a single clutch during one of these peaks was 32. In the laboratory, under highly favorable conditions, the time between mating and releasing young can be as short as 4 weeks (Morritt & Stevenson, 1993). Combining the latter two observations, we set *R*_m_ = 32 offspring per month. We have no survival estimates of our population and used the observation that daily mortality percentage, *m*, is 0.9% per day in *O. gammarellus* in southern Portugal (Dias & Sprung, 2003) to create a dummy data set where the proportion survivors, *s*, at day *t* + 1 was related to the proportion survivors at day *t* and *m* following $s_{t+1}=s_{t}-m\cdot s_{t}$, with *m* = 0.9% and *s*_0_ = 1. Using this dummy dataset, we estimated the monthly mortality rate by estimating the slope of the regression of log‐transformed proportion survivors [month^‐1^] against time [month] at *µ* = 0.27 month^‐1^ (see Smallegange & Berg, 2019). We also do not have observations on the von Bertalanffy growth rate $\overset{\cdot }{\underset{B}{r}}$ and instead used the observed estimated growth rate for *O. gammarellus* females in southern Portugal at 1.52/year (Dias & Sprung, 2003), which, expressed per month, equals $\overset{\cdot }{\underset{B}{r}}=1.52/1/12=0.13$ month^‐1^. It is likely that the von Bertalanffy growth rate and mortality rate differ between populations at our field site and in southern Portugal. However, our perturbation analysis (see Section 3.3, Figure 4) revealed that *L*_p_ and *L*_m_ were most influential to the log stochastic population growth rate, from which we infer that model outcomes are highly insensitive to the specific values of the von Bertalanffy growth rate and mortality rate. Finally, we assumed $E_{L_{B}}\left(L\left(t\right)\right)=L_{B}=3.80$ mm and $\sigma_{L_{B}}^{2}\left(L\left(t\right)\right)=0.001$, and, for the starvation condition, we assumed the commonly used value of *κ* = 0.80 (Add‐my‐pet, 2016). All parameter values are presented in Table 1; note that the time step *t* of the DEB‐IPM is 1 month for *O. gammarellus*. A model performance check is presented in Smallegange and Berg (2019).

**Table 1:** DEB parameters for female *Orchestia gammarellus* in a population on the island of Schiermonnikoog, The Netherlands and for female *Manta alfredi* in a population of Yaeyama Islands, Japan (Kashiwagi, 2014)

Symbol	Description	*O. gammarellus*		*M. alfredi*	
		Value	Unit	Value	Unit
*E*(*Y*)	Expected feeding level at which DEB parameters are measured	NA	—	NA	—
*L*_b_	Body length at birth	3.79	mm	130	cm
*L*_p_	Body length at puberty (maturity)	7.29	mm	380	cm
$L_{\infty }$	Ultimate length	$L_{m}\cdot E\left(Y\right)$	mm	NA	cm
*L*_m_	Maximum length at *E*(*Y*) = 1	15.61	mm	550	cm
*R*_m_	Maximum reproduction rate at *L*_m_	32	# month^−1^	1	# year^−1^
$\overset{\cdot }{r_{B}}$	von Bertalanffy growth rate	0.13	month^−1^	0.18	year^−1^
*κ*	Energy allocation fraction to somatic maintenance and growth	0.80	—	0.80	—
*µ*	Mortality rate	0.27	month^−1^	0.05	year^−1^
*σ*(*Y*)	Standard deviation of expected feeding level used in simulations	0.1 or 0.5	—	0.1 or 0.5	—

Note

For *O. gammarellus*, parameters were estimated using data from our field populations, except for $\overset{\cdot }{r_{B}}$ and* µ*, which were estimated for a population of *O. gammarellus* in southern Portugal (Dias & Sprung, 2003).

Abbreviation: NA, not available.

### 2.4 DEB‐IPM parameterization for the reef manta ray

The reef manta ray *M. alfredi* (Mobulidae) (Figure 1b) is one of the largest rays in the world and is distributed worldwide in tropical and subtropical waters. They are nonmigratory and live close to coasts, reefs or islands, where they aggregate at cleaning stations and areas where they feed on zooplankton (Marshall et al., 2011). We chose to use the DEB‐IPM with the additional assumption that individuals cannot shrink under starvation conditions (as shrinking is less likely to occur in vertebrates than in invertebrates) (Smallegange et al., 2017). We use published life history data on female reef manta rays obtained from a stable population off the coasts of Yaeyama Islands, Japan, which population growth rate is estimated at 1.02–1.03 per year (Kashiwagi, 2014). We use the disk width, that is, the distance between the two pectoral fin tips, as the measure for body length. Length at birth is measured at 130 cm and females mature at about 10 years of age at a minimum length of 380 cm (Kashiwagi, 2014) and live at least 40 years, reaching a maximum length of 550 cm (Marshall et al., 2011). On average, adult females produce one pup every 2 years (Kashiwagi, 2014), but this can be as high as one pup every year (Marshall et al., 2011). Based on these observations, we set *L*_b_ = 130 cm, *L*_p_ = 380 cm, *L*_m_ = 550 cm and *R*_m_ = 1/year. The survival rate of juveniles and adults is estimated at 0.95 (Kashiwagi, 2014) and we set the mortality rate constant at $\mu =-\log\left(0.95\right)=0.05/\text{year}.$ The von Bertalanffy growth rate is estimated for females at $\overset{\cdot }{\underset{B}{r}}$ = 0.18 (Kashiwagi, 2014). We assumed $E_{L_{B}}\left(L\left(t\right)\right)=L_{B}=130$ cm and $\sigma_{L_{B}}^{2}\left(L\left(t\right)\right)=0$. For the starvation condition, we assumed the commonly used value of *κ* = 0.80 (Add‐my‐pet, 2016). All data are summarized in Table 1; note that the time step *t* of the DEB‐IPM is 1 year for the reef manta ray.

### 2.5 Stochastic demographic model

We used the stochastic demographic model $p\left(t+1\right)=A\left(t\right)\cdot p\left(t\right)$, where **p**(*t*) is the population vector at time *t* and **A**(*t*) is a DEB‐IPM at time *t* defined by a two‐state Markov chain that gives the probability distribution of environment states at time *t*. In this chain, state 1 is the good environment and state 2 is the bad environment. This results in the following Markov chain habitat transition matrix **H** (Caswell, 2001, p. 379):

(8) $$H=\left[\begin{array}{cc} 1-p & q\\ p & 1-q \end{array}\right],$$

where *p* is the probability of switching from the good to the bad environment, and *q* is the probability of switching from the bad to the good environment. The serial or autocorrelation of the Markov chain equals *ρ* = 1 − *p* − *q* (Caswell, 2001, p. 379). High, positive values of *ρ* are referred to as red noise; high and negative values of *ρ* as blue noise; and *ρ* = 0 denotes white noise where the probability of switching states is independent of the current state. The temporal frequency at which the good environment occurs is given by *f* = *q*/(*p* + *q*) (Caswell, 2001, p. 379). We used a high feeding level $E{\left(Y\right)}_{\text{high}}$ and a low feeding level $E{\left(Y\right)}_{\text{low}}$ to define the good and bad environmental states, respectively. Specifically, above and below the feeding level where we predict population equilibrium at *λ* = 1, we defined $E{\left(Y\right)}_{\text{low}}$ as the expected feeding level associated with *λ* where reproduction was negligible and defined $E{\left(Y\right)}_{\text{high}}$ as the expected feeding level associated with a very high value of *λ*. For *O. gammarellus*, this implied the values $E{\left(Y\right)}_{\text{low}}=0.4$ and $E{\left(Y\right)}_{\text{high}}=1.0$ (Smallegange & Berg, 2019), and for the reef manta ray values were, respectively, below and above the expected feeding level of $E\left(Y\right)=0.75$ for which we previously predicted population equilibrium at *λ* = 1 (Smallegange et al., 2017): $E{\left(Y\right)}_{\text{low}}=0.50$ and $E{\left(Y\right)}_{\text{high}}=0.90$. By iterating **H** (for all combinations of *p* and *q*) a time series of length 3,000 (with an initial transient length of 500 discarded, a starting population of one individual in each size bin, and with the initial environment state chosen randomly [see also Tuljapurkar, Horvitz, & Pascarella, 2003]) was generated (Smallegange et al., 2014). This sequence determines the environment state, and hence the feeding level *E*(*Y*), that a population experiences at each time step, from which the individual‐level functions were calculated to construct **A**(*t*), that is, the DEB‐IPM at time *t* defined by the feeding level *E*(*Y*) at time *t*. At each time step, **A**(*t*) was stored with associated vectors of population structure to calculate the log of the stochastic growth rate *λ*_s_ as $\log\left(\lambda_{s}\right)=\frac{1}{\tau }\sum_{\tau =0}^{\tau -1}r_{t}$ with $r_{t}=\log\left[p\left(t+1\right)/p\left(t\right)\right]$, where *τ* = 3,000 − 500 = 2,500. At each time step, also the mean of the body size distribution was calculated, after which we used the pooled mean body size, calculated as the grand mean of all mean body sizes over time period* τ*, for our analysis. To explore to what extent demographic stochasticity affects patterning, we varied the standard deviation of the expected feeding level, σ(*Y*), and ran all analyses first with σ(*Y*) = 0.1, and then with *σ*(*Y*) = 0.5.

### 2.6 Perturbation analysis

We conducted a perturbation analysis to examine the elasticity of *λ*_s_ to perturbation of each of the model parameters *L_b_*, *L_p_*, *L_m_*, *R*_m_, $\overset{\cdot }{\underset{B}{r}}$, and *µ* (Table 1) to identify which life history parameters under which stochastic regimes were most influential to the long‐run, log stochastic population growth rate log(*λ*_s_). For this, we perturbed each parameter by 1% and calculated the elasticity of log(*λ*_s_) to each model parameter. Again, we varied the standard deviation of the expected feeding level, *σ*(*Y*), and ran the perturbation analysis first with *σ*(*Y*) = 0.1, and then with *σ*(*Y*) = 0.5.

We excluded the parameter* κ*, because, apart from occurring in the starvation condition (Equation 2), *κ* is also related to *L*_m_ (which is mathematically proportional to* κ*) and *R*_m_ (which is mathematically proportional to [1 − *κ*]) (Kooijman & Metz, 1984). A perturbation analysis of *κ* is, therefore, mathematically challenging and biological interpretation of its results difficult as effects of variation in *κ* will carry over to affect survival, growth as well as reproduction. Finally, *κ* is the only parameter that cannot be measured directly on individuals, which would make it impractical as an indicator of population performance.

## 3 RESULTS

### 3.1 Shifts in log(*λ*_s_) across stochastic environments

**Figure 2:**
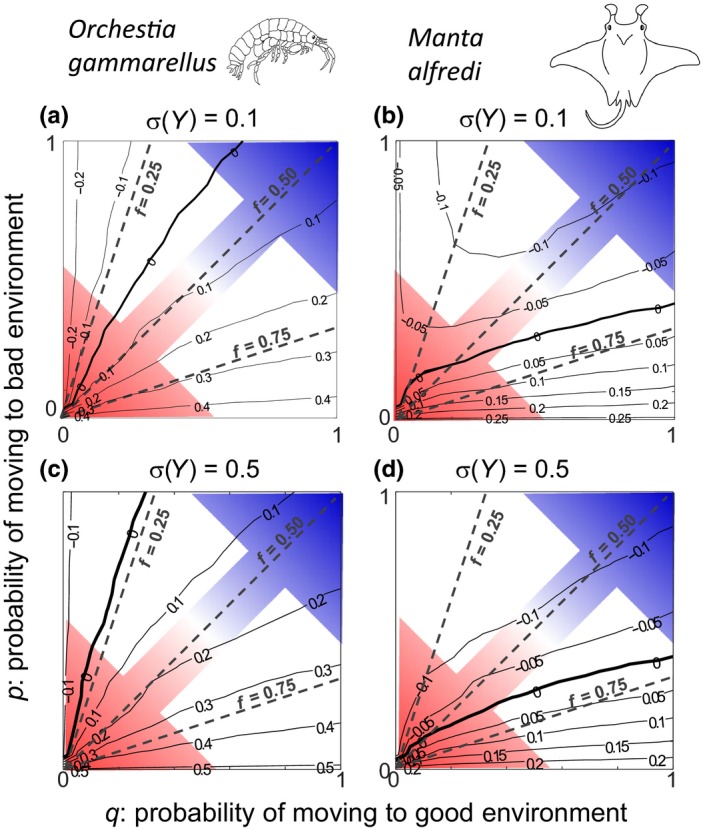
Shifts in log(*λ*_s_) across stochastic environments when variability in expected feeding level is low, (a) and (b), or high, (c) and (d). Contour plots show for *Orchestia gammarellus* (a) and (c) and *Manta alfredi* (b) and (d) how the log stochastic population growth rate (log(*λ*_s_)) relates to the probability of switching from the bad to good environment (*q*) and the probability of switching from the good to bad environment (*p*) depending on variability in expected feeding level. The thick solid line denotes population equilibrium at log(*λ*_s_) = 0. The colored arrows show values of *p* and *q* where the autocorrelation (*ρ*) in environmental regimes is red (bottom‐left‐hand corner), white (antidiagonal) and blue (top‐right‐hand corner); the three dashed lines in each panel show values of *p* and *q* in which the good environment frequency *f* equals 0.25, 0.50 and 0.75. For *O. gammarellus*, $E{\left(Y\right)}_{\text{low}}=0.4$ and $E{\left(Y\right)}_{\text{high}}=1.0$; for *M. alfredi*, $E{\left(Y\right)}_{\text{low}}=0.5$ and $E{\left(Y\right)}_{\text{high}}=0.9$

Under low variability in expected feeding level (*σ*(*Y*) = 0.1), our stochastic analysis revealed for *O. gammarellus* that, across all combinations of the probability of switching from the bad to good environment (*q*), and the probability of switching from the good to bad environment (*p*), increases in log(*λ*_s_) closely matched increases in the frequency of good environments (*f*) (Figure 2a: solid lines denoting constant values of log(*λ*_s_) are closely parallel to dashed lines denoting constant values of *f*). Therefore, at intermediate values around *f* = 0.5, shifts in noise color, that is, the autocorrelation *ρ*, moving from red noise at the bottom‐left part of Figure 2a to blue noise at the top‐right part of Figure 2a, has little impact on log(*λ*_s_) (Figure 2a: solid line of log(*λ*_s_) = 0.1 is closely parallel to *f* = 0.5). In contrast, at low values of *f*, log(*λ*_s_) increases slightly as noise color shifts from red to blue, whereas at high values of *f*, log(*λ*_s_) decreases slightly as noise color shifts from red to blue (Figure 2a). For *M. alfredi*, a different pattern emerged. Like *O. gammarellus*, log(*λ*_s_) increased with increasing *f*, but the correlation was less strong, particularly at low values of *f* (Figure 2b: solid lines of constant log(*λ*_s_) are much less parallel to dashed lines of constant *f*). In contrast to *O. gammarellus*, log(*λ*_s_) always decreased as noise colored shifted from red to blue (Figure 2b: moving along the arrow from red to blue, log(*λ*_s_) always decreases); the magnitude of this decrease, however, increased with increasing values of *f*.

Under high variability in expected feeding level (*σ*(*Y*) = 0.5), very similar results emerged (Figure 2c,d). The most notable difference was that log(*λ*_s_) of *O. gammarellus* was higher for each combination of *p* and *q* if *σ*(*Y*) = 0.5 than if *σ*(*Y*) = 0.1 (Figure 2c vs. 2a). Variability in expected feeding level had little effect on log(*λ*_s_) of *M. alfredi* (Figure 2d vs. 2b). Log(*λ*_s_) was overall slightly lower under high compared to low variability in expected feeding level (Figure 2d vs. 2b), and log(*λ*_s_) levels off to a low plateau under white/blue noise and low values of *f* (top‐left corner of Figure 2d) if σ(*Y*) = 0.5, whereas if σ(*Y*) = 0.1, log(*λ*_s_) values reached a shallow valley in that same region (top‐left corner of Figure 2b).

### 3.2 Shifts in mean body size across stochastic environments

**Figure 3:**
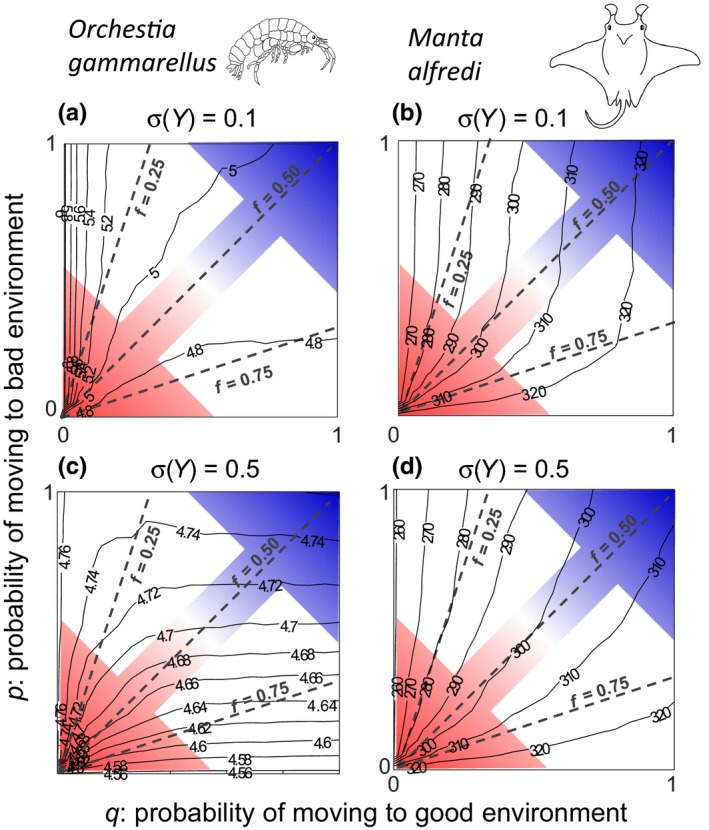
Shifts in pooled mean body size across stochastic environments when variability in expected feeding level is low, (a) and (b), or high, (c) and (d). Contour plots show how pooled mean body size (mm) for *Orchestia gammarellus*, (a) and (c), and pooled mean body size (cm) for *Manta alfredi*, (b) and (d), the stochastic population growth rate (log(*λ*_s_)) relates to the probability of switching from the bad to good environment (*q*) and the probability of switching from the good to bad environment (*p*) depending on variability in expected feeding level. The colored arrows show values of *p* and *q* where the autocorrelation (*ρ*) in environmental regimes is red (bottom‐left‐hand corner), white (antidiagonal) and blue (top‐right‐hand corner); the three dashed lines in each panel show values of *p* and *q* in which the good environment frequency *f* equals 0.25, 0.50 and 0.75. For *O. gammarellus*, $E{\left(Y\right)}_{\text{low}}=0.4$ and $E{\left(Y\right)}_{\text{high}}=1.0$; for *M. alfredi*, $E{\left(Y\right)}_{\text{low}}=0.5$ and $E{\left(Y\right)}_{\text{high}}=0.9$

Under low variability in expected feeding level (*σ*(*Y*) = 0.1), pooled mean body size for *O. gammarellus* decreased with increasing values of the frequency of good environments (*f*) (Figure 3a: solid lines denoting constant values of mean body size were close to being parallel to dashed lines denoting constant values of *f*). This is likely due to an increase in the number of smaller individuals (eggs, juveniles) as also log(*λ*_s_) increased with increasing values of *f* (Figure 2a). Variation in noise color had some effect on pooled mean body size of *O. gammarellus* (Figure 3a): for low values of *f*, mean body size decreased when moving from red to blue noise, whereas the opposite pattern occurred at high values of *f*. In contrast to *O. gammarellus*, pooled mean body size of *M. alfredi* increased with increasing values of the frequency of good environments (*f*), particularly under red noise (Figure 3b). Like *O. gammarellus*, variation in noise color only had a small effect on pooled mean body size, which increased slightly when moving from red to blue noise for all but the highest values of *f* (Figure 3b). Interestingly, under certain conditions, the probability of moving to the bad environment (*p*), has little effect on pooled mean body size of both species. For *O. gammarellus* this was the case when values of *q*, the probability of moving to the good environment, were low. For *M. alfredi*, this was the case for a broad range of *q* values, as long as value of *p* was not low (Figure 3b).

Under high variability in expected feeding level (σ(*Y*) = 0.5), pooled mean body size for *O. gammarellus* was overall smaller than under low variability in expected feeding level (Figure 3c vs. 3a); again this matches the finding that log(*λ*_s_) was overall higher under high than low variability in expected feeding level (Figure 2c vs. 2a), such that increased population growth leads to proportionally more smaller individuals (eggs, juveniles) in the population. Unlike under low variability in expected feeding level, pooled mean body size was insensitive to shifts in *q*, the probability of moving to the good environment, for almost all values of *p*, the probability of moving to the bad environment. Pooled mean body size slightly increased when moving from red to blue noise at higher values of *f* (Figure 3c). For *M. alfredi*, pooled mean body size was overall smaller under low variability in expected feeding level (Figure 3d vs. 3b).

### 3.3 Perturbation analysis

**Figure 4:**
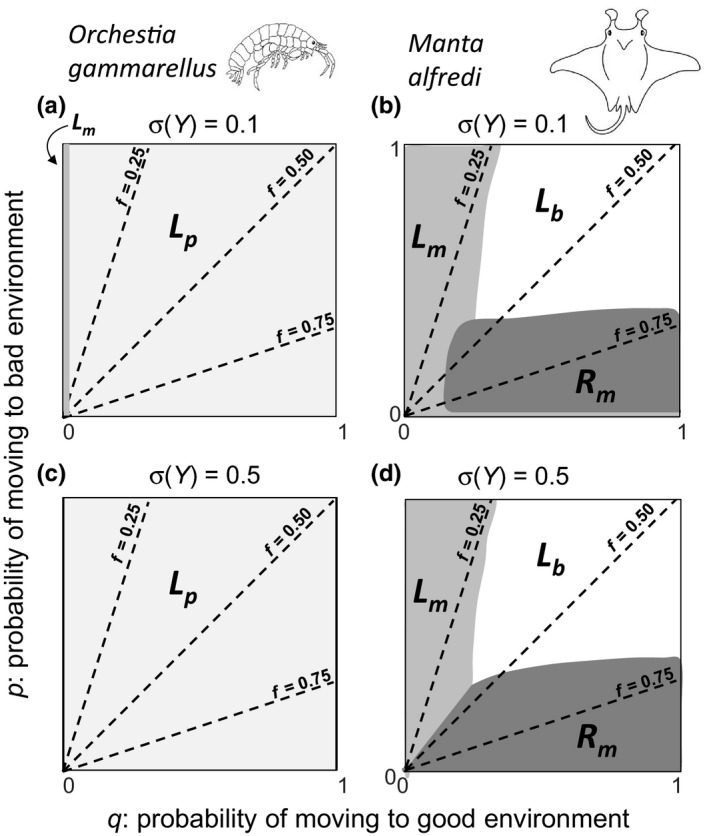
Perturbation analysis results when variability in expected feeding level is low, (a) and (b), or high, (c) and (d). In case of *Orchestia gammarellus*, the stochastic population growth rate (log(*λ*_s_)) was nearly always most sensitive to perturbation of length at puberty (*L*_p_) (light gray areas) (a) and (c), except for a small region at very low values of *q*, the probability of moving from the bad to the good environment, and low variability in expected feeding level, where log(*λ*_s_) was most sensitive to perturbation of maximum length (*L*_m_) (dark gray areas) (a). In case of *Manta alfredi*, log(*λ*_s_), was either most sensitive to perturbation of *L_m_*, length at birth (*L*_b_) or maximum reproduction rate (*R*_m_) (b) and (d), which patterns differed slightly under red noise and higher values of *f* (bottom‐left corner of b and d). The three dashed lines in each panel show values of *p* and *q* in which the good environment frequency *f* equals 0.25, 0.50 and 0.75. For *O. gammarellus*, $E{\left(Y\right)}_{\text{low}}=0.4$ and $E{\left(Y\right)}_{\text{high}}=1.0$; for *M. alfredi*, $E{\left(Y\right)}_{\text{low}}=0.5$ and $E{\left(Y\right)}_{\text{high}}=0.9$

Under low variability in expected feeding level (σ(*Y*) = 0.1), for *O. gammarellus*, the perturbation analysis revealed that across nearly all values of *p* and *q*, perturbation of *L_p_* elicited the maximum elasticity values of log(*λ*_s_) (light gray area in Figure 4a), except for *q* = 0, where perturbation of maximum length, *L*_m_, elicited the highest elasticity values (dark gray area in Figure 4a). For *M. alfredi*, log(*λ*_s_) was most sensitive to, respectively, perturbation of *L*_m_, length at birth *L*_b_, and maximum reproduction rate, *R*_m_, with increasing values of *f* (Figure 4b). Under red noise, log(*λ*_s_) was most sensitive to perturbation of *L*_m_ or *R*_m_.

Under high variability in expected feeding level (σ(*Y*) = 0.5), perturbation results were very similar. For *O. gammarellus*, the only difference was that for all values of *p* and *q*, the stochastic population growth rate was always most sensitive to perturbation of length at maturity (Figure 4c). For *M. alfredi*, a larger region under red noise and higher values of *f* (bottom‐left corner of Figure 4d) was now most sensitive to perturbation of *R*_m_ instead of *L*_m_ (Figure 4d vs. 4b).

## 4 DISCUSSION

Our goal was to examine the functional pathways by which a typical fast and a typical slow life history species differ in their response to shifts in temporal autocorrelation when their individual growth and reproduction are described by functional, life history traits. We chose our study species such that this study is also a demonstration of how a unified method like the DEB‐IPM can be applied to extremely different species. The reef manta ray *M. alfredi* (slow life history) consistently responded to a shift from red to blue noise with a decrease in stochastic population growth rate, regardless of the frequency at which good environments occurred. In contrast, the stochastic population growth rate of *O. gammarellus* (fast life history) either decreased or increased in response to a shift from red to blue noise, depending on whether the good environment frequency was high or low. More significantly, *O. gammarellus* stochastic population growth rate was more sensitive to shifts in good environment frequency than to shifts in temporal autocorrelation of food conditions: the higher the frequency value, the higher was the stochastic population growth rate. Qualitatively equivalent patterns emerged when the same stochastic analysis was applied to another fast life history species, the bulb mite *Rhizoglyphus robini* (Smallegange & Ens, 2018).

### 4.1 Life history patterns across stochastic environments

Our findings on life history speed sensitivity to shifts in environmental autocorrelation from a mechanistic analysis are opposite to the recent outcome of the phenomenological, cross‐taxonomical analysis by Paniw et al. (2018). Although we only have stochastic functional trait analysis outcomes for three species (this study; Smallegange & Ens, 2018), we find there is merit in discussing potential reasons for why the two sets of results differ. Such a discussion is also urgent in light of the persistent problems that exist in the construction of standard matrix population models (Kendall et al., 2019), such that Kendall et al. (2019) advocate to be cautious when interpreting results from, for example, cross‐taxonomical analyses that use such models. To start, it is important to note that the finding by Paniw et al. (2018) that fast life histories were on average more sensitive to environmental autocorrelation than slow species only explained 50% of the variance in sensitivity to autocorrelation. Our study species could therefore simply fall into the category of species that drive the unexplained variance. However, they might not in which case other explanations should be considered that focus on the difference in model approach. Standard stochastic analyses of matrix models perturb demographic rates directly to explore population consequences (Boyce et al., 2006; Morris et al., 2008; Paniw et al., 2018). Because demographic rates in most such matrix models are statistical descriptors (with a mean and variance) of empirical observations (but see Klanjscek, Caswell, Neubert, & Nisbet, 2006), they lack a mechanistic underpinning of how environmental variation impacts demography through individual life history trajectories. An automatic assumption of such an model approach is that statistical descriptors of demographic rates apply to all explored environments (elsewhere we explain what problems can arise if this assumption is not justified; Smallegange et al., 2017). In a mechanistic approach, like ours, expected feeding level within a stochastic food time series defines the state of the current environment, which subsequently determines an individual's growth and reproduction as dependent on its body size, and an energy allocation trade‐off (Smallegange et al., 2017). This presence versus absence of a mechanistic description of demography could explain the contrasting outcomes of our stochastic functional trait analysis and of standard stochastic analyses (Paniw et al., 2018). Further analyses are clearly warranted to confirm or reject this hypothesis, but we may need to be cautious with regard to the assumed mechanisms of how environmental variation affects demography. It also signifies the potential importance of integrating functional traits into demographic models (Salguero‐Gómez et al., 2018). The fact that in our functional trait approach species only differed in life history trait values, and not in model structure as is often the case in standard stochastic analyses (Paniw et al., 2018), should facilitate future cross‐taxonomical comparative studies to evaluate which functional traits best describe and predict life history patterns and population dynamics across variable environments.

Interpreting a population's response to shifts in environmental patterning requires detailed knowledge of the variation in underlying demographic rates (Ezard & Coulson, 2010; Morris & Doak, 2004). Our perturbation analysis allows for a functional analysis of how environmental change affects population responses, which revealed that the fast life history species *O. gammarellus* was most sensitive to perturbation of length at puberty: the body length at which individuals start reproducing. The higher the feeding level, the quicker an individual reaches its length at puberty resulting in higher reproduction, to which the stochastic population growth rate responds the strongest. Of the two quantities that defined the environmental patterning in our simulations—good environment frequency and noise color—good environment frequency directly determines experienced feeding levels. Noise color, instead, is associated with both low and high good environment frequencies. It is therefore not surprising that a fast life history species responds more strongly to shifts in good environment frequency, as this directly affects its experienced feeding level, and thus reproductive output. The slow life history species *M. alfredi* was instead predominantly most sensitive to perturbation of two life history parameters important for growth: body length at birth and maximum body length. Only in very favorable environments, characterized by high good environment frequencies, was *M. alfredi* stochastic population growth rate most sensitive to a life history parameter associated with reproduction (maximum reproduction rate). Perhaps because *M. alfredi* was more sensitive to perturbation of growth parameters, it is less sensitive to shifts in good environment frequency, and, as a corollary, more sensitive to shifts in noise color. A stochastic functional trait analysis of species from across the full fast‐slow life history continuum would reveal whether the relative influence of variation in good environment frequency and noise color indeed shifts across the slow‐fast life history continuum. Particularly for threatened species, this is important to ascertain, as these typically have slow life histories. Whereas standard stochastic analysis outcomes show that slow life history species are mostly buffered against shifts in temporal autocorrelation (Paniw et al., 2018), our functional trait analysis of one slow life history species reveals that its persistence could be affected by shifts in noise color, such as those of climate variables that are ongoing right now (García‐Carreras & Reuman, 2011).

### 4.2 Body size as population performance indicator

Understanding how populations and communities respond to shifts in environmental patterning and how they can transition from stability to decline is an ongoing challenge in ecology (Carpenter et al., 2011; Folke et al., 2004; Scheffer et al., 2009). The newest indices of population performances to predict such switches in population state include information on the dynamics of phenotypic traits (Baruah, Clements, Guillaume, & Ozgul, 2019), such as body size (Clements & Ozgul, 2016). However, despite many descriptive patterns of how trait and population dynamics relate (Baruah et al., 2019; Clements & Ozgul, 2016), we know little about how life history processes drive body size as an indicator of population performance. For the fast life history species *O. gammarellus*, we find that shifts in stochastic population growth rate across variable environments are correlated with shifts in body size distribution resulting from variation in reproductive effort. Specifically, reproduction is high (or low) in favorable (or unfavorable) environments, increasing (or decreasing) the relative abundance of eggs and juveniles, which shifts the body size distribution toward smaller (or larger) sizes, affecting the population growth rate. For the slow life history species *M. alfredi*, shifts in body size distribution were also related to shifts in stochastic population growth rate, albeit more loosely and in a different manner, as population growth rate was most sensitive to perturbation of growth parameters and not reproduction. As environments become less favorable (or unfavorable) across the stochastic gradient, *M. alfredi* showed decreased (or increased) individual growth that shifts the body size distribution toward smaller (or larger) sizes, decreasing (or increasing) population growth rate. Such downward shifts in mean body size have been observed across a range of taxa in response to adverse environmental change (Gardner, Peters, Kearney, Joseph, & Heinsohn, 2011; Sheridan & Bickford, 2011), which has carried over to, for example, reduce survival rates in red knots *Calidris canutus canutus* (van Gils et al., 2016), and alter the shape of the density‐dependence function in Soay sheep *Ovis aries* (Coulson et al., 2008). All in all, our results support the recent proposition that shifts in body size can be indicative of a shift in a population's performance (Clements & Ozgul, 2016). In addition, in the case of ecosystem engineers, such as the soil bioturbator *O. gammarellus*, shifts in body size distribution can have direct effects on ecosystem functioning, as the impact of its bioturbation depends upon body size distributions and associated population size (Schrama, Boheemen, Olff, & Berg, 2015). It will be interesting to investigate how large a plastic or genetic shift in life history trait values should be to counter any negative effects of environmental change signaled by a shifts in body size distribution.

### 4.3 Demographic stochasticity

Finally, we investigated the role of demographic stochasticity in all responses to shifts in environmental quality patterning. Demographic stochasticity has for a long time been ignored as its impact on the long‐term population growth rate levels off with population size (May, 1973). Recent insights, however, highlight how demographic variance, population structure and temporal autocorrelation of environmental conditions can interact to shape population dynamics (Vindenes & Engen, 2017). We found that an increase in demographic stochasticity elicited opposite responses for our fast and slow life history species. In case of *O. gammarellus*, pooled mean body size decreased (due to a proportional increase in small individuals) with increasing demographic stochasticity, whereas that of *M. alfredi* decreased (likely due to decreased growth), all else being equal. As a result, the stochastic population growth rate increased with increasing demographic stochasticity for *O. gammarellus* but decreased for *M. alfredi*. Given the fact that the stochastic population growth rate of each species is sensitive to other life history parameters, this could explain why a change in the variance in expected feeding level that fuels the demographic stochasticity, elicited difference responses, as it affects the life histories differently. An increase in demographic stochasticity also elicited a shift in the sensitivity of mean body size to the probabilities with which environments switched quality state. At low demographic stochasticity, mean body size of the slow life history species *M. alfredi*, and to a lesser extent that of the fast life history *O. gammarellus*, was mostly insensitive to shifts in the probability of moving to the bad environment. At high demographic stochasticity, the fast life history *O. gammarellus* was mostly insensitive to shifts in the probability of moving to the good environment, whereas mean body size of *M. alfredi* was now sensitive to shifts in both probabilities. We do not know what the underlying cause is of these differences between the two species. However, given the important role of body size in population dynamics (De Roos & Persson, 2013), the resilience of food webs to disturbance (Woodward et al., 2005), and even the strength of trophic cascades (DeLong et al., 2015), investigating the life history mechanisms behind how species of different life history respond to shifts in demographic stochasticity plays will be a valuable effort.

## CONFLICT OF INTEREST

None declared.

## AUTHORS' CONTRIBUTION

Isabel Smallegange: conception and design, construction of the population model and performance of the simulations, interpretation of the results, writing of the paper. Matty Berg: conception and design, collection and interpretation of field data, contributed to the interpretation of the results, the writing of the paper, review, and edition of the manuscript prior to submission.

## Data Availability

Field data, model parameters, simulation code and the *Orchestia gammarellus* model check to create the figures are stored on Figshare: https://doi.org/10.6084/m9.figshare.6795371 (Smallegange & Berg, 2019).
